# Methyl 3-(pyridin-4-yl­methyl­idene)di­thio­carbazate

**DOI:** 10.1107/S1600536813009409

**Published:** 2013-04-13

**Authors:** Hui Wang, Abul-Monsur-Showkot Hossain, Shi-Chao Wang, Yu-Peng Tian

**Affiliations:** a Deparment of Chemistry, Anhui University, Hefei 230039, People’s Republic of China; b Key Laboratory of Functional Inorganic Materials, Chemistry, Hefei 230039, People’s Republic of China

## Abstract

There are two independent mol­ecules in the asymmetric unit of the title mol­ecule, C_8_H_9_N_3_S_2_, both of which exhibit an *E* conformation with the pyridine ring and di­thio­carbazate fragment located on opposite sides of the C=N bond. The pyridine ring and di­thio­carbazate group are approximately coplanar, with dihedral angles of 4.74 (1) and 8.77 (1)° between their planes in the two mol­ecules. In the crystal, mol­ecules are linked to each other *via* N—H⋯N hydrogen bonds, forming zigzag chains parallel to [10-1].

## Related literature

For related structures, see: Shan *et al.* (2006 ▶); Chen *et al.* (2007 ▶). Derivatives of the title compound are often used as coordinating ligands in the metal complexes, see for example: Wu *et al.* (2001 ▶); Fun *et al.* (2001 ▶).
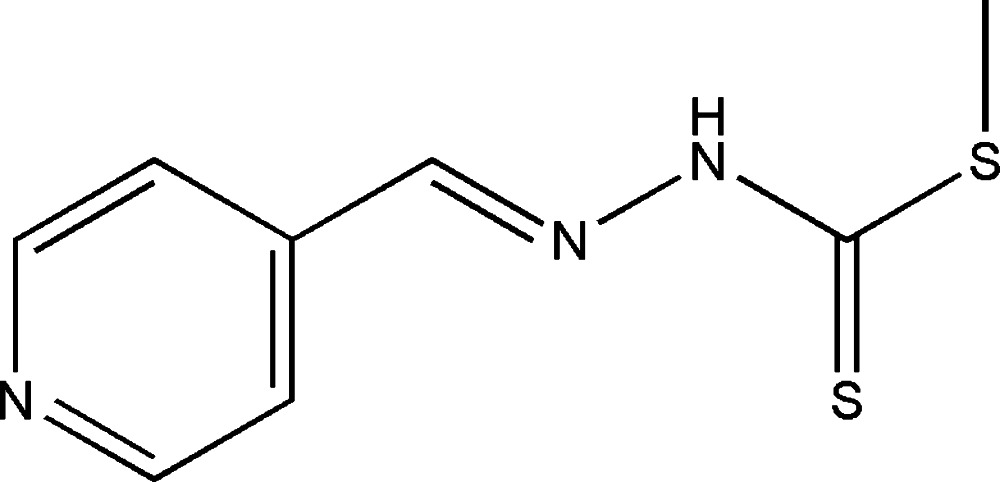


## Experimental

### 

#### Crystal data

C_8_H_9_N_3_S_2_
*M* *_r_* = 211.30
Monoclinic, 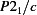

*a* = 7.547 (5) Å
*b* = 20.216 (5) Å
*c* = 13.415 (5) Å
β = 96.070 (5)°
*V* = 2035.3 (16) Å^3^
*Z* = 8
Mo *K*α radiation
μ = 0.48 mm^−1^
*T* = 296 K
0.30 × 0.20 × 0.20 mm

#### Data collection

Bruker SMART CCD area-detector diffractometer
Absorption correction: multi-scan (*SADABS*; Bruker, 2002 ▶) *T*_min_ = 0.870, *T*_max_ = 0.910
14118 measured reflections
3565 independent reflections
2379 reflections with *I* > 2σ(*I*)
*R*_int_ = 0.035

#### Refinement

*R*[*F* ^2^ > 2σ(*F* ^2^)] = 0.044
*wR*(*F* ^2^) = 0.124
*S* = 1.02
3565 reflections
237 parameters
H-atom parameters constrained
Δρ_max_ = 0.23 e Å^−3^
Δρ_min_ = −0.40 e Å^−3^

### 

Data collection: *SMART* (Bruker, 2002 ▶); cell refinement: *SAINT* (Bruker, 2002 ▶); data reduction: *SAINT*; program(s) used to solve structure: *SHELXS97* (Sheldrick, 2008 ▶); program(s) used to refine structure: *SHELXL97* (Sheldrick, 2008 ▶); molecular graphics: *SHELXTL* (Sheldrick, 2008 ▶); software used to prepare material for publication: *SHELXTL*.

## Supplementary Material

Crystal structure: contains datablock(s) I, global. DOI: 10.1107/S1600536813009409/gg2112sup1.cif

Structure factors: contains datablock(s) I. DOI: 10.1107/S1600536813009409/gg2112Isup2.hkl

Supplementary material file. DOI: 10.1107/S1600536813009409/gg2112Isup3.cml

Additional supplementary materials:  crystallographic information; 3D view; checkCIF report

## Figures and Tables

**Table 1:** Hydrogen-bond geometry (Å, °)

*D*—H⋯*A*	*D*—H	H⋯*A*	*D*⋯*A*	*D*—H⋯*A*
N3*B*—H3*B*⋯N1*A* ^i^	0.86	2.04	2.904 (3)	179
N3*A*—H3*A*⋯N1*B* ^ii^	0.86	2.07	2.909 (3)	166

Symmetry codes: (i) 
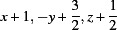
; (ii) 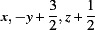.

**Fig. 1.**
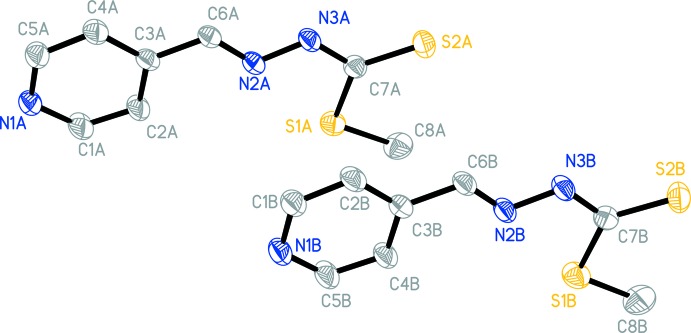
: The molecular structure of (I), with the atom-numbering scheme. Displacement ellipsoids are drawn at the 30% probability level. H atoms have been omitted.

**Fig. 2.**
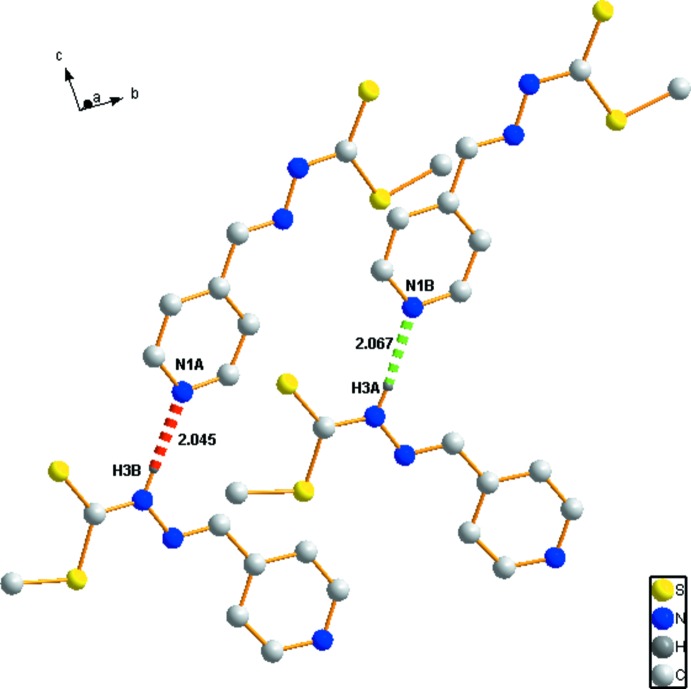
: The H-bond diagram of the title molecule (I).

**Fig. 3.**
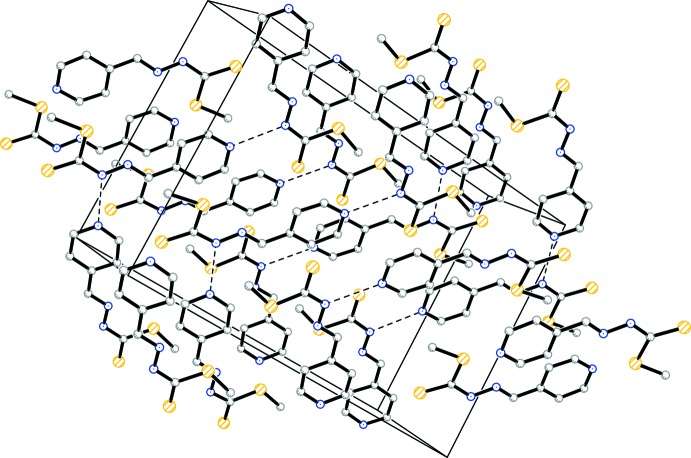
A packing diagram.

## supplementary crystallographic information

### Comment

The derivatives of the title compound, (I), are often used as coordinating ligands in the metal complexes (Wu *et al.*, 2001; Fun *et al.* 2001). Herewith, in this study, we report the crystal structure of the title compound (I). The dithiocarbazate moiety shows an E configuration about the C(6A)—N(2A) and N(3A)—C(7A) bonds.Through planar as whole, the molecules comprise two planar fragments, namely the pyridine moiety and dithiocarbazate moiety with dihedral angles of 4.74 (1)° and 8.77 (1)°, respectively.The bond distances of C(7A)—S(2 A) and C(7A)—S(1 A) are different compared to the bond lengths of C(7B)—S(2B) and C(7B)—S(1B). So the crystal structures of the two molecules are independent. The value for the C=S bond of two molecules is almost same with corresponding C=S bond of related compounds. Also, the pairs of centrosymmetrically related molecules are linked into dimers by pairs of N(1A)···H(3B) and N(1B)···H(3A) hydrogen bonds.

### Experimental

A hot solution of *S*-Methyldithiocarbazate(0.488 mg, 4 mmol) in ethanol (30 mL) was mixed with a 4-formylpyridine (0.535 mg, 5 mmol) in ethanol 10 mL and the reaction mixture was reflux. After one hour, precipitated was appeared. Under cooling at room temperature the light yellow crystals were separated by filtration and recrystallized from methanol. Yield: 70%. ^1^H NMR (400 MHz, DMSO-d^6^) 13.5 (s, 1H), 8.6 (d, 2H), 8.2 (d, 2H), 7.6 (s, 2H), 2.5 (s, 3H).

### Refinement

All hydrogen atoms were placed in geometrically idealized positions and constrained to ride on their parent atoms, with C—H = 0.93 Å and *U*_iso_(H) = 1.2 *U*_eq_.

### Crystal data

**Table:**

C_8_H_9_N_3_S_2_	*F*(000) = 880
*M**_r_* = 211.30	*D*_x_ = 1.379 Mg m^−^^3^
Monoclinic, *P*2_1_/*c*	Mo *K*α radiation, λ = 0.71069 Å
Hall symbol: -P 2ybc	Cell parameters from 3068 reflections
*a* = 7.547 (5) Å	θ = 3.1–23.7°
*b* = 20.216 (5) Å	µ = 0.48 mm^−^^1^
*c* = 13.415 (5) Å	*T* = 296 K
β = 96.070 (5)°	Needle, yellow
*V* = 2035.3 (16) Å^3^	0.30 × 0.20 × 0.20 mm
*Z* = 8	

### Data collection

**Table:**

Bruker SMART CCD area-detector diffractometer	3565 independent reflections
Radiation source: fine-focus sealed tube	2379 reflections with *I* > 2σ(*I*)
Graphite monochromator	*R*_int_ = 0.035
phi and ω scans	θ_max_ = 25.0°, θ_min_ = 1.8°
Absorption correction: multi-scan (*SADABS*; Bruker, 2002)	*h* = −8→8
*T*_min_ = 0.870, *T*_max_ = 0.910	*k* = −24→23
14118 measured reflections	*l* = −15→15

### Refinement

**Table:**

Refinement on *F*^2^	Primary atom site location: structure-invariant direct methods
Least-squares matrix: full	Secondary atom site location: difference Fourier map
*R*[*F*^2^ > 2σ(*F*^2^)] = 0.044	Hydrogen site location: inferred from neighbouring sites
*wR*(*F*^2^) = 0.124	H-atom parameters constrained
*S* = 1.02	*w* = 1/[σ^2^(*F*_o_^2^) + (0.0609*P*)^2^ + 0.3327*P*] where *P* = (*F*_o_^2^ + 2*F*_c_^2^)/3
3565 reflections	(Δ/σ)_max_ = 0.002
237 parameters	Δρ_max_ = 0.23 e Å^−^^3^
0 restraints	Δρ_min_ = −0.40 e Å^−^^3^

### Special details

**Table:**

Geometry. All e.s.d.'s (except the e.s.d. in the dihedral angle between two l.s. planes) are estimated using the full covariance matrix. The cell e.s.d.'s are taken into account individually in the estimation of e.s.d.'s in distances, angles and torsion angles; correlations between e.s.d.'s in cell parameters are only used when they are defined by crystal symmetry. An approximate (isotropic) treatment of cell e.s.d.'s is used for estimating e.s.d.'s involving l.s. planes.
Refinement. Refinement of *F*^2^ against ALL reflections. The weighted *R*-factor *wR* and goodness of fit *S* are based on *F*^2^, conventional *R*-factors *R* are based on *F*, with *F* set to zero for negative *F*^2^. The threshold expression of *F*^2^ > σ(*F*^2^) is used only for calculating *R*-factors(gt) *etc*. and is not relevant to the choice of reflections for refinement. *R*-factors based on *F*^2^ are statistically about twice as large as those based on *F*, and *R*- factors based on ALL data will be even larger.

### Fractional atomic coordinates and isotropic or equivalent isotropic displacement parameters (Å^2^)

**Table:**

	*x*	*y*	*z*	*U*_iso_*/*U*_eq_	
S1A	0.35871 (10)	0.85984 (3)	0.66511 (5)	0.0612 (2)	
S2A	0.61864 (10)	0.86303 (4)	0.85370 (5)	0.0664 (2)	
S2B	1.13863 (10)	1.12665 (4)	0.84421 (6)	0.0682 (2)	
S1B	0.88859 (11)	1.12370 (4)	0.65218 (6)	0.0701 (3)	
N2A	0.4094 (3)	0.72614 (10)	0.67557 (13)	0.0506 (5)	
N3A	0.5016 (2)	0.75677 (10)	0.75618 (13)	0.0517 (5)	
H3A	0.5605	0.7337	0.8023	0.062*	
N2B	0.9044 (3)	0.98971 (10)	0.67700 (14)	0.0511 (5)	
C7A	0.4990 (3)	0.82288 (12)	0.76252 (17)	0.0497 (6)	
C3A	0.3291 (3)	0.62711 (12)	0.58701 (17)	0.0465 (6)	
N1A	0.1530 (3)	0.55737 (10)	0.42552 (15)	0.0577 (6)	
C3B	0.8138 (3)	0.89050 (12)	0.59250 (17)	0.0495 (6)	
C6B	0.9048 (3)	0.92688 (12)	0.67716 (17)	0.0510 (6)	
H6B	0.9630	0.9040	0.7312	0.061*	
N1B	0.6448 (3)	0.82087 (11)	0.42879 (15)	0.0637 (6)	
N3B	0.9971 (2)	1.02071 (10)	0.75677 (14)	0.0520 (5)	
H3B	1.0445	0.9982	0.8071	0.062*	
C4B	0.7214 (4)	0.92342 (13)	0.51193 (17)	0.0644 (8)	
H4B	0.7152	0.9694	0.5108	0.077*	
C7B	1.0131 (3)	1.08713 (12)	0.75522 (18)	0.0509 (6)	
C6A	0.4216 (3)	0.66340 (12)	0.67161 (16)	0.0488 (6)	
H6A	0.4892	0.6406	0.7225	0.059*	
C4A	0.3405 (3)	0.55944 (12)	0.58017 (18)	0.0585 (7)	
H4A	0.4080	0.5356	0.6300	0.070*	
C2B	0.8164 (3)	0.82227 (13)	0.58876 (18)	0.0572 (7)	
H2B	0.8757	0.7982	0.6412	0.069*	
C2A	0.2238 (4)	0.65942 (14)	0.51115 (17)	0.0662 (8)	
H2A	0.2105	0.7051	0.5123	0.079*	
C5A	0.2517 (3)	0.52680 (13)	0.49947 (18)	0.0609 (7)	
H5A	0.2615	0.4810	0.4967	0.073*	
C1A	0.1395 (4)	0.62239 (14)	0.4342 (2)	0.0717 (9)	
H1A	0.0677	0.6447	0.3845	0.086*	
C1B	0.7311 (3)	0.78998 (13)	0.50717 (18)	0.0613 (7)	
H1B	0.7337	0.7440	0.5067	0.074*	
C5B	0.6397 (4)	0.88672 (14)	0.4341 (2)	0.0726 (9)	
H5B	0.5764	0.9093	0.3815	0.087*	
C8B	0.9334 (4)	1.20963 (13)	0.6749 (3)	0.0900 (10)	
H8B	0.9026	1.2213	0.7402	0.135*	
H7B	0.8639	1.2356	0.6252	0.135*	
H9B	1.0578	1.2181	0.6713	0.135*	
C8A	0.3883 (4)	0.94601 (13)	0.6945 (2)	0.0754 (8)	
H8A	0.3611	0.9539	0.7618	0.113*	
H9A	0.3101	0.9718	0.6487	0.113*	
H10A	0.5096	0.9584	0.6887	0.113*	

### Atomic displacement parameters (Å^2^)

**Table:**

	*U*^11^	*U*^22^	*U*^33^	*U*^12^	*U*^13^	*U*^23^
S1A	0.0645 (5)	0.0584 (4)	0.0575 (4)	0.0059 (3)	−0.0086 (3)	0.0075 (3)
S2A	0.0795 (5)	0.0582 (4)	0.0575 (4)	−0.0075 (4)	−0.0124 (4)	−0.0068 (3)
S2B	0.0697 (5)	0.0607 (5)	0.0707 (5)	−0.0100 (3)	−0.0088 (4)	−0.0095 (3)
S1B	0.0829 (6)	0.0631 (5)	0.0609 (5)	0.0095 (4)	−0.0075 (4)	0.0092 (3)
N2A	0.0549 (13)	0.0558 (13)	0.0379 (11)	−0.0022 (10)	−0.0093 (9)	0.0007 (9)
N3A	0.0597 (14)	0.0523 (12)	0.0392 (11)	0.0006 (10)	−0.0129 (10)	0.0002 (9)
N2B	0.0565 (13)	0.0553 (13)	0.0388 (11)	−0.0006 (10)	−0.0083 (9)	−0.0028 (9)
C7A	0.0487 (15)	0.0537 (15)	0.0460 (14)	−0.0015 (11)	0.0021 (11)	0.0010 (11)
C3A	0.0478 (14)	0.0561 (15)	0.0340 (13)	−0.0002 (11)	−0.0023 (11)	0.0018 (10)
N1A	0.0720 (15)	0.0527 (13)	0.0446 (12)	−0.0026 (11)	−0.0108 (11)	−0.0016 (10)
C3B	0.0528 (15)	0.0558 (16)	0.0383 (14)	−0.0007 (12)	−0.0029 (11)	0.0011 (11)
C6B	0.0562 (16)	0.0571 (16)	0.0368 (13)	0.0012 (12)	−0.0083 (11)	0.0010 (11)
N1B	0.0824 (16)	0.0600 (15)	0.0444 (12)	−0.0048 (12)	−0.0140 (11)	−0.0041 (10)
N3B	0.0574 (13)	0.0534 (13)	0.0418 (11)	0.0034 (10)	−0.0104 (10)	−0.0024 (9)
C4B	0.091 (2)	0.0529 (16)	0.0439 (15)	−0.0012 (14)	−0.0171 (14)	0.0027 (12)
C7B	0.0501 (15)	0.0536 (15)	0.0490 (14)	0.0044 (12)	0.0050 (12)	0.0012 (11)
C6A	0.0543 (15)	0.0515 (15)	0.0376 (13)	0.0002 (12)	−0.0084 (11)	0.0036 (11)
C4A	0.0710 (18)	0.0518 (16)	0.0479 (15)	0.0079 (13)	−0.0163 (13)	0.0040 (12)
C2B	0.0698 (18)	0.0577 (17)	0.0402 (14)	0.0054 (13)	−0.0121 (12)	0.0042 (11)
C2A	0.096 (2)	0.0478 (15)	0.0481 (15)	0.0069 (14)	−0.0244 (15)	0.0006 (12)
C5A	0.077 (2)	0.0478 (16)	0.0533 (16)	0.0030 (13)	−0.0130 (14)	−0.0013 (11)
C1A	0.096 (2)	0.0611 (18)	0.0502 (16)	0.0082 (15)	−0.0296 (16)	0.0030 (13)
C1B	0.081 (2)	0.0523 (16)	0.0481 (16)	0.0012 (13)	−0.0059 (14)	−0.0005 (12)
C5B	0.106 (2)	0.0561 (18)	0.0479 (16)	0.0010 (15)	−0.0256 (16)	0.0024 (13)
C8B	0.111 (3)	0.0575 (18)	0.101 (3)	0.0072 (18)	0.008 (2)	0.0194 (17)
C8A	0.085 (2)	0.0578 (17)	0.082 (2)	0.0140 (15)	0.0053 (17)	0.0112 (15)

### Geometric parameters (Å, º)

**Table:**

S1A—C7A	1.759 (2)	N1B—C5B	1.334 (3)
S1A—C8A	1.795 (3)	N3B—C7B	1.348 (3)
S2A—C7A	1.654 (2)	N3B—H3B	0.8600
S2B—C7B	1.650 (3)	C4B—C5B	1.372 (3)
S1B—C7B	1.751 (2)	C4B—H4B	0.9300
S1B—C8B	1.790 (3)	C6A—H6A	0.9300
N2A—C6A	1.273 (3)	C4A—C5A	1.379 (3)
N2A—N3A	1.370 (2)	C4A—H4A	0.9300
N3A—C7A	1.339 (3)	C2B—C1B	1.375 (3)
N3A—H3A	0.8600	C2B—H2B	0.9300
N2B—C6B	1.270 (3)	C2A—C1A	1.376 (3)
N2B—N3B	1.368 (2)	C2A—H2A	0.9300
C3A—C4A	1.375 (3)	C5A—H5A	0.9300
C3A—C2A	1.386 (3)	C1A—H1A	0.9300
C3A—C6A	1.465 (3)	C1B—H1B	0.9300
N1A—C1A	1.324 (3)	C5B—H5B	0.9300
N1A—C5A	1.329 (3)	C8B—H8B	0.9600
C3B—C2B	1.380 (3)	C8B—H7B	0.9600
C3B—C4B	1.392 (3)	C8B—H9B	0.9600
C3B—C6B	1.462 (3)	C8A—H8A	0.9600
C6B—H6B	0.9300	C8A—H9A	0.9600
N1B—C1B	1.332 (3)	C8A—H10A	0.9600
			
C7A—S1A—C8A	101.42 (13)	C3A—C4A—C5A	120.0 (2)
C7B—S1B—C8B	101.51 (14)	C3A—C4A—H4A	120.0
C6A—N2A—N3A	116.77 (19)	C5A—C4A—H4A	120.0
C7A—N3A—N2A	119.44 (19)	C1B—C2B—C3B	119.7 (2)
C7A—N3A—H3A	120.3	C1B—C2B—H2B	120.1
N2A—N3A—H3A	120.3	C3B—C2B—H2B	120.1
C6B—N2B—N3B	117.1 (2)	C1A—C2A—C3A	118.6 (3)
N3A—C7A—S2A	121.75 (18)	C1A—C2A—H2A	120.7
N3A—C7A—S1A	112.84 (17)	C3A—C2A—H2A	120.7
S2A—C7A—S1A	125.41 (15)	N1A—C5A—C4A	123.4 (2)
C4A—C3A—C2A	117.1 (2)	N1A—C5A—H5A	118.3
C4A—C3A—C6A	121.5 (2)	C4A—C5A—H5A	118.3
C2A—C3A—C6A	121.5 (2)	N1A—C1A—C2A	124.9 (3)
C1A—N1A—C5A	116.0 (2)	N1A—C1A—H1A	117.5
C2B—C3B—C4B	117.2 (2)	C2A—C1A—H1A	117.5
C2B—C3B—C6B	121.6 (2)	N1B—C1B—C2B	123.7 (3)
C4B—C3B—C6B	121.2 (2)	N1B—C1B—H1B	118.2
N2B—C6B—C3B	120.1 (2)	C2B—C1B—H1B	118.2
N2B—C6B—H6B	120.0	N1B—C5B—C4B	124.5 (3)
C3B—C6B—H6B	120.0	N1B—C5B—H5B	117.7
C1B—N1B—C5B	116.1 (2)	C4B—C5B—H5B	117.7
C7B—N3B—N2B	118.9 (2)	S1B—C8B—H8B	109.5
C7B—N3B—H3B	120.5	S1B—C8B—H7B	109.5
N2B—N3B—H3B	120.5	H8B—C8B—H7B	109.5
C5B—C4B—C3B	118.7 (3)	S1B—C8B—H9B	109.5
C5B—C4B—H4B	120.7	H8B—C8B—H9B	109.5
C3B—C4B—H4B	120.7	H7B—C8B—H9B	109.5
N3B—C7B—S2B	121.11 (18)	S1A—C8A—H8A	109.5
N3B—C7B—S1B	113.02 (18)	S1A—C8A—H9A	109.5
S2B—C7B—S1B	125.87 (15)	H8A—C8A—H9A	109.5
N2A—C6A—C3A	120.0 (2)	S1A—C8A—H10A	109.5
N2A—C6A—H6A	120.0	H8A—C8A—H10A	109.5
C3A—C6A—H6A	120.0	H9A—C8A—H10A	109.5
			
C6A—N2A—N3A—C7A	177.6 (2)	C4A—C3A—C6A—N2A	179.6 (2)
N2A—N3A—C7A—S2A	−175.66 (17)	C2A—C3A—C6A—N2A	−1.5 (4)
N2A—N3A—C7A—S1A	4.1 (3)	C2A—C3A—C4A—C5A	1.1 (4)
C8A—S1A—C7A—N3A	−179.21 (18)	C6A—C3A—C4A—C5A	180.0 (2)
C8A—S1A—C7A—S2A	0.5 (2)	C4B—C3B—C2B—C1B	0.6 (4)
N3B—N2B—C6B—C3B	−177.8 (2)	C6B—C3B—C2B—C1B	−179.2 (2)
C2B—C3B—C6B—N2B	177.9 (2)	C4A—C3A—C2A—C1A	−0.6 (4)
C4B—C3B—C6B—N2B	−1.8 (4)	C6A—C3A—C2A—C1A	−179.5 (3)
C6B—N2B—N3B—C7B	174.2 (2)	C1A—N1A—C5A—C4A	−1.6 (4)
C2B—C3B—C4B—C5B	−0.2 (4)	C3A—C4A—C5A—N1A	0.1 (4)
C6B—C3B—C4B—C5B	179.5 (3)	C5A—N1A—C1A—C2A	2.2 (5)
N2B—N3B—C7B—S2B	−174.34 (17)	C3A—C2A—C1A—N1A	−1.1 (5)
N2B—N3B—C7B—S1B	6.0 (3)	C5B—N1B—C1B—C2B	−2.0 (4)
C8B—S1B—C7B—N3B	178.32 (18)	C3B—C2B—C1B—N1B	0.6 (4)
C8B—S1B—C7B—S2B	−1.3 (2)	C1B—N1B—C5B—C4B	2.4 (5)
N3A—N2A—C6A—C3A	−179.1 (2)	C3B—C4B—C5B—N1B	−1.3 (5)

### Hydrogen-bond geometry (Å, º)

**Table:**

*D*—H···*A*	*D*—H	H···*A*	*D*···*A*	*D*—H···*A*
N3*B*—H3*B*···N1*A*^i^	0.86	2.04	2.904 (3)	179
N3*A*—H3*A*···N1*B*^ii^	0.86	2.07	2.909 (3)	166

Symmetry codes: (i) *x*+1, −*y*+3/2, *z*+1/2; (ii) *x*, −*y*+3/2, *z*+1/2.
